# Amino acid side chain contribution to protein FTIR spectra: impact on secondary structure evaluation

**DOI:** 10.1007/s00249-021-01507-7

**Published:** 2021-02-08

**Authors:** Joëlle De Meutter, Erik Goormaghtigh

**Affiliations:** Laboratory for the Structure and Function of Biological Membranes, Center for Structural Biology and Bioinformatics, Université Libre de Bruxelles, Campus Plaine CP206/02, 1050 Brussels, Belgium

**Keywords:** FTIR spectroscopy, Secondary structure, Protein spectroscopy, Protein microarrays, Amino acid side chain

## Abstract

Prediction of protein secondary structure from FTIR spectra usually relies on the absorbance in the amide I–amide II region of the spectrum. It assumes that the absorbance in this spectral region, i.e., roughly 1700–1500 cm^−1^ is solely arising from amide contributions. Yet, it is accepted that, on the average, about 20% of the absorbance is due to amino acid side chains. The present paper evaluates the contribution of amino acid side chains in this spectral region and the potential to improve secondary structure prediction after correcting for their contribution. We show that the β-sheet content prediction is improved upon subtraction of amino acid side chain contributions in the amide I–amide II spectral range. Improvement is relatively important, for instance, the error of prediction of β-sheet content decreases from 5.42 to 4.97% when evaluated by ascending stepwise regression. Other methods tested such as partial least square regression and support vector machine have also improved accuracy for β-sheet content evaluation. The other structures such as α-helix do not significantly benefit from side chain contribution subtraction, in some cases prediction is even degraded. We show that co-linearity between secondary structure content and amino acid composition is not a main limitation for improving secondary structure prediction. We also show that, even though based on different criteria, secondary structures defined by DSSP and XTLSSTR both arrive at the same conclusion: only the β-sheet structure clearly benefits from side chain subtraction. It must be concluded that side chain contribution subtraction benefit for the evaluation of other secondary structure contents is limited by the very rough description of side chain absorbance which does not take into account the variations related to their environment. The study was performed on a large protein set. To deal with the large number of proteins present, we worked on protein microarrays deposited on BaF_2_ slides and FTIR spectra were acquired with an imaging system.

**Supplementary Information:**

The online version contains supplementary material available at 10.1007/s00249-021-01507-7.

## Introduction

Fourier transform infrared (FTIR) spectroscopy has become a global tool for the study of protein structure (Barth 2007; Wang et al. 2008), protein glycans (Derenne et al. 2020) and lipids (Dreissig et al. 2009; Derenne et al. 2014). Numerous publications report the establishment of a mathematical relationship between protein FTIR spectra and secondary structure content (Lee et al. 1990; Prestrelski et al. 1992; Pribic et al. 1993; Severcan et al. 2001; Smith et al. 2002; Oberg et al. 2003, 2004; Wilcox et al. 2016). To improve protein secondary structure prediction from FTIR spectra, we recently defined a large reference protein set, called cSP92, containing 92 commercially available proteins for which high-resolution structures are available (De Meutter and Goormaghtigh 2020) and we investigated the potential of partial deuteration to improve secondary structure prediction (De Meutter and Goormaghtigh 2021a) This protein set was designed to span the entire structural space in terms of secondary structures and also higher-order structures as described by CATH (Orengo et al. 1997). Great care was taken to check that the proteins used in the experimental work have the same origin/sequence as the protein from which the high-resolution structure was obtained. Finally, the purity of the proteins retained in cSP92 and used in the present experimental work was checked by SDS-PAGE.

Protein secondary structure prediction is usually based on the analysis of the amide I and amide II band of proteins. The mathematical process implicitly assumes that the absorbance in this spectral region, i.e., roughly 1700–1500 cm^−1^, is solely arising from amide contributions. Yet, it is accepted that, on the average, about 20% of the absorbance in this region is due to amino acid side chains (Chirgadze et al. 1975; Rahmelow et al. 1998) and this value varies obviously from protein to protein according to its primary structure. Evaluating the contribution of amino acid side chains in the amide I–amide II spectral region is, therefore, of utmost interest. There are numerous examples in the literature of analyses of some specific side chain for understanding how enzyme work, e.g., (Barth 2002; Barth and Zscherp 2002; Liu et al. 2005; Lórenz-Fonfría et al. 2015; Rudack et al. 2015; Kottke et al. 2017) but there is much less work reporting systematic information on all side chains. The first extensive series of data was reported by Chirgadze et al. (1975) and Venyaminov and Kalnin (1991), reviewed in Goormaghtigh et al. (1994a). These data have the advantage to express side chain contributions as a sum of Lorentzian/Gaussian bands whose parameters (band position, width, intensity, fraction of Gaussian component) are known. It is, therefore, easy to synthetize these contributions for each protein as a function of the actual amino acid content. Numerous papers have added information to this first set of data as reviewed extensively by Barth (2000, 2007). More recently, another extensive study of the infrared spectra and molar absorption coefficient of the 20 amino acid side chains was reported by Wolpert and Hellwig (2006). The latter data were obtained on free amino acids and do not therefore include the effect for a side chain being included in a peptide. Yet, it remains one of the most extensive sources of consistent information on side chain absorption. At the opposite, Ramelow et al. (1998) included amino acids in short peptides and extracted mathematically the side chain contributions. Due to experimental limitations, results are not as detailed as for free amino acids but have the advantage of considering the side chains in a peptidic environment. Numerous papers also report theoretical computations of side chain contributions. There are extremely valuable to better understand the spectra and to test the effect of the environment on the spectrum. For instance, a series of such computations has been carried out in Ghomi’s lab (Derbel et al. 2007; Hernández et al. 2009, b, 2010a; Pflüger et al. 2010). Yet, results cannot be directly used to process experimental data as frequencies, scaling and band widths are not precisely known.

The key issue for the purpose of this work is that the actual amino acid side chain contributions in the amide I–amide II spectral region depends on many parameters. Besides physical parameters such as temperature (Anderson et al. 2014) or pressure (Scott and Vanderkooi 2011), most variability comes from the environment of the side chain including H-bonds, local pH, local polarity of the medium, coupling with other vibrations (Bagchi et al. 2009), etc. which cannot be accessed nor taken properly into account. Furthermore, the expected ionization may be strongly affected by modification of pK_a_ such as in bacteriorhodopsin where Asp^96^ has a pK_a_ above 12 (Zscherp et al. 1999). It can, therefore, not be expected that an exact subtraction of amino acid side chain contribution can be obtained. We address in this paper the question of the potential secondary structure prediction improvement that could be achieved by subtracting a rough estimate of the side chain contributions in the amide I–amide II spectral range.

To deal with the large number of proteins present in cSP92, we recently proposed to work on protein microarrays deposited on BaF_2_ slides. Protein sample density is in the range of 2,000–4,000 samples per cm^2^ (De Meutter et al. 2016, 2017) and FTIR spectra are acquired with an imaging system. This approach results in fast measurement and high signal-to-noise ratio spectra. It has been applied here to obtain all FTIR spectra presented.

## Materials and methods

### Proteins and protein secondary structure content

The list of the proteins, their commercial source and their characterization (sequence, purity, etc.) has been reported earlier (De Meutter and Goormaghtigh 2020). The high-resolution structure PDB files were obtained for each protein from the PDB repository (Bernstein et al. 1977). A list of the selected PDB files for each protein can be found in De Meutter and Goormaghtigh (2020). Each PDB file was analyzed by DSSP (Kabsch and Sander 1983) which defines 8 secondary structure types: H, α-helix content denoted here dH to indicate it has been obtained according to DSSP definition; dE, β-sheet; dG, 3_10_-helix; dI, π-helix; dT, helix-turn; dB, β-bridge and dS, bend. The amino acids that do not belong to one of these structures are assigned to d-. In this work, we also define whatever is not α-helix or β-sheet as “dOthers”, i.e. dOthers = 100-dH-dE. All values are expressed in % of the total number of amino acids present in a protein.

Another set of secondary structure definitions has also been tested. XTLSSTR (King and Johnson 1999) has been initially designed for the analysis of circular dichroism (CD) spectra. It identifies xH, α-helix; xE, β-strand (E); xG, 3_10_-helix; xT, hydrogen-bonded turn; xN, non-hydrogen-bonded turn and xP, poly(L-proline) II type 3_1_-helix. It also makes a distinction between the core of the α-helix “H” and the side or unconnected stretch “h”. The sum xH + xh is, therefore, the total α-helix content. It similarly makes a difference between xE and xe. Overall, large discrepancies can be observed between dH and xH or dE and xE as the definitions are different (De Meutter and Goormaghtigh 2021c).

All structural features have been extracted and tabulated from the PDB files and the DSSP files output files by a module of the home-made Kinetics software running under Matlab, as described elsewhere (De Meutter and Goormaghtigh 2020).

### Protein microarrays printing

Details of the experimental procedure are described elsewhere (De Meutter et al. 2016, 2017). Microarrays were printed with an Arrayjet Marathon noncontact inkjet Microarrayer (ArrayJet, Roslin, UK) on 40 × 26 × 2 mm^3^ BaF_2_ slides (Neyco, France). Drops of ca. 100 pL protein solution were deposited to form regular arrays. Spot diameter was about 80 μm. Spot-to-spot distances in the *X* and *Y* directions were 200 μm or 220 µm, resulting in ca 2,000 protein samples per cm^2^.

### FTIR imaging of protein microarrays

FTIR imaging of protein microarrays has been described earlier (De Meutter et al. 2016, 2017). Briefly spectra were recorded as the average of 64 scans/pixel, between 3650 and 900 cm^−1^ at a nominal resolution of 8 cm^−1^. FTIR data were collected using an Agilent mid-IR imager equipped with a liquid nitrogen cooled 128 × 128 Mercury Cadmium Telluride (MCT) Focal Plane Array (FPA) detector and a 15X objective (NA = 0.62). Automated spectrum extraction was described previously (De Meutter et al. 2017), including the procedure followed to subtract the background. A single spot usually contained ca 300 pixels, i.e., 300 spectra. After correction for background, spectra filtered for signal-to-noise ratio and maximum absorbance were averaged (De Meutter et al. 2016, 2017). Finally, the average spectra of quadruplicates obtained for a same protein were averaged, yielding one spectrum per protein. Spectra were then baseline-corrected by subtraction of a straight line interpolated between the spectral points at 1720 and 1480 cm^−1^. Scaling was obtained by dividing the spectra by the area under the spectrum between 1720 and 1480 cm^−1^.

### Subtraction of amino acid side chain contribution

Side chain contributions, in first approximation represented as the sum of Lorentzian–Gaussian bands, can be easily reconstituted (Goormaghtigh et al. 1996; Raussens et al. 2004; Goormaghtigh 2009). The key issue is the use of realistic parameters to represent the side chain absorption as discussed under Results and Discussion. Briefly, each vibration mode requires at least 4 parameters: band intensity, band position, band width and fraction of Gaussian. Usually one side chain contribution is made out of several such bands. For instance, in the amide I–amide II spectral range, arginine has a strong absorption near 1673 cm^−1^ assigned to ν_as_(CN_3_H_5_^+^), a less strong one near 1633 cm^−1^ assigned to ν_s_(CN_3_H_5_^+^) and a weaker one at 1522 cm^−1^ assigned to δ_s_(CN_3_H_5_^+^). In such a case, the three contributions have to be summed up to obtain the contribution of arginine. In other instances, different ionization states have to be taken into account. For instance, aspartic acid has a major band at 1729 cm^−1^ assigned to ν (C=O) for the protonated form of the carboxylic acid and a major band at 1570 cm^−1^ for the ionized form assigned to ν_as_(COO^−^). In such a case, the experimental pH and the pK_a_ are taken into account to compute the fractional contribution of both forms which are then summed up. Finally, all the side chain contributions are added to obtain the overall contribution of the side chains that will be ultimately subtracted from the protein spectrum. The parameters used to compute the side chain contributions are reported in Table S1.

### Secondary structure prediction from FTIR spectra

The mathematical relation between FTIR spectra and secondary structure content has been established as described earlier (De Meutter and Goormaghtigh 2021b). Briefly the ascending stepwise linear regression (ASLR) introduces, in an ascending stepwise manner, one absorbance at a time in a linear regression model (Goormaghtigh et al. 2006, 2009). Partial least square regression (PLS) is a multivariate approach that minimizes the number of latent variables (LVs) required for prediction (Geladi and Kowalski 1986; Wold et al. 2001). It was computed by the software running under Matlab developed by Norgaart et al. (Nørgaard et al. 2000; Leardi and Nørgaard 2005). Support Vector Machine (SVM) dedicated to solving regression problems (Tange et al. 2015; Ghorbani et al. 2016) was used according to the formulation introduced by Suykens et al., with the Matlab toolbox built by the authors (Pelckmans et al. 2002).

Two types of validations were obtained. Cross-validation was run in a leave-one-out mode, i.e., one protein spectrum at a time was removed from the training set and used to challenge the model obtained with all the other proteins. The quality of the prediction was computed as the root mean square standard error in cross-validation (RMSECV). This error was compared with the standard deviation of the secondary structure content (STDDEV^REFCV^) by computing ζ^CV^ = STDDEV^REFCV^/RMSECV (Oberg et al. 2004; Kinalwa et al. 2010). ζ indicates how much better the prediction is with respect to guessing the mean values is the prediction. For instance, a value of ζ = 3 for the α-helix whose content distribution in cSP92 is characterized by STDDEV^REFCV^ = 18.3% means that the error of prediction is 6.1%. When ζ is close to 1, it indicates spectroscopy does not bring much added value to secondary structure prediction. It must be noted that ζ is related to the correlation coefficient (Fearn 2002).

A second calibration used a single subset of the cSP92 protein spectra as test set. The Kennard–Stone algorithm (Kennard and Stone 1969) was used to select one third of the spectra with a uniform distribution of the secondary structure content. The quality of the prediction was judged from the root mean square error of prediction for the Kennard–Stone selected test set (RMSEKS) and ζ^KS^ was computed as STDDEV^REFKS^/RMSEKS. It must be noted that STDDEV^REFCV^ is different from STDDEV^REFKS^.

### Computations

Image analysis, spectrum processing, subtraction of side chain contributions and multivariate analyses were all performed with Kinetics, a home-made software running under MatLab (The MathWorks Inc.).

## Results and discussion

### Description of amino acid side chain bands

It must be kept in mind that it remains difficult to select the best parameters describing amino acid side chain absorption as they vary very significantly as a function of the environment. Side chain contributions used in this work will be described as an overlap of Lorentzian–Gaussian bands (Goormaghtigh et al. 1996; Raussens et al. 2004). Amino acid side chain band parameters have been reported in H_2_O (Venyaminov and Kalnin 1991) and D_2_O (Chirgadze et al. 1975) and extensively used to correct FTIR spectra of proteins in the course of ^1^H/^2^H exchange (Raussens et al. 1997, 2004; Meskers et al. 1999). Yet, while working with cSP92 protein spectra, it was noticed that the subtracted contribution does not always match the shape required to correct the ^1^H form of the protein spectra. In this paper we introduce the data provided by Wolpert and Hellwig (2006) and revised some these values in view of the data provided by Rahmelow et al. (1998) as indicated in Table S1. The missing elements, usually the band width, were taken from Venyaminov and Kalnin (Venyaminov and Kalnin 1991). All band parameters are provided in Table S1. Table S1 highlights the modifications brought to the original data published by Wolpert and Hellwig. With these parameters, resulting corrected spectra appeared to be of better quality but subtraction of two side chains, Glu and Tyr, remained problematic, i.e., the position of the band was obviously shifted with respect to their actual contribution in cSP92 protein spectra, resulting in local negative deviation in the corrected spectra. For this reason, it was of interest to investigate whether cSP92 protein spectra themselves could be a source of information.

### Extracting amino acid side chain contributions from protein FTIR spectra

In principle, the 92 spectra of the cSP92 protein set absorbance matrix (between 1720 and 1480 cm^−1^) **A** can be tentatively expressed as a linear combination of concentrations (**C** matrix) and “pure” spectra (**S** matrix) for the amide bonds and amino acid side chains, **A** = **S.C**. The **C** matrix contains the content in dH, dE, dOthers and in the 9 amino acids which have a significant contribution in the amide I–amide II spectral range (see list in Table S1). All concentration values were obtained from the DSSP output files as described in Materials and Methods. The **S** matrix can therefore be obtained by simple matrix division. Such an approach had been suggested by Rahmelow et al. (1998) for a series of short peptides, usually tripeptides, including the different amino acids.

**Fig. 1:**
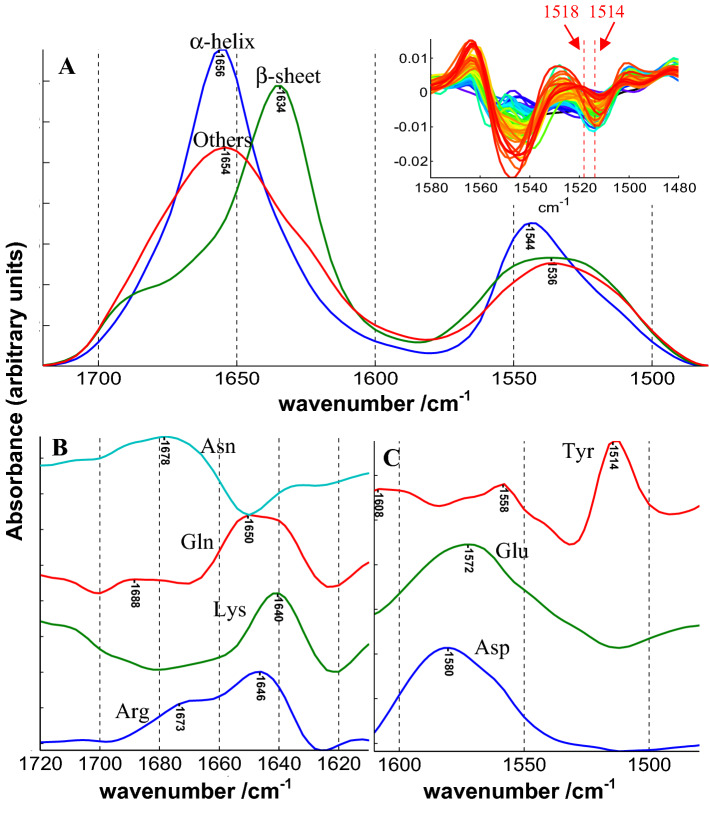
Shape of the different components contributing to the FTIR spectrum of cSP92 proteins in the amide I–amide II spectral region. The **C** matrix contains for each protein the concentration in dH, dE, dOthers, Asp, Glu, Tyr, Gln, Asn, Arg and Lys. Other amino acids absorb here but the intensity of their contribution is minor. **a** Shape of the α-helix (dH), β-sheet (dE) and the “Others” structures (dOthers). **b** Shape of the amino acid whose main contribution is found the amide I region of the spectrum. **c** shape of the contribution of amino acids whose main contribution is found in the amide II region of the spectrum. The inset in part A. of the figure reports the second derivative between 1580 and 1480 cm^−1^ of the protein spectra of cSP92 and the two red vertical lines report the observed tyrosine ring vibration at 1514 and the expected value for free Tyr amino acid at 1518 cm^−1^

Extracting **S** is obviously a more difficult enterprise here as the variance in the concentrations is limited, some co-linearity is present (see later and Fig. S1) and there is no unique pure component perfectly representing each secondary structure or amino acid side chain. Indeed, the spectrum of each secondary structure or side chain present important variations related to the environment. This variability can obviously not be realistically reflected in a unique spectrum. Yet, keeping these limitations in mind, it is interesting to examine the shape of the pure components present in **S** obtained by matrix division. Results are reported in Fig. 1. It is surprising to extract for the α-helices (dH), β-sheet (dE) and the “Others” structure spectra that exactly match the expectations. Both the rather narrow amide I band centered at 1656 cm^−1^ together with amide II centered on 1544 cm^−1^ are characteristic features of the α-helix. The much broader amide I band centered at 1654 together with amide II at 1536 cm^−1^ structures are the exact features expected for the “Others” which mainly contains the disordered part of protein structure. The same comment stands for the β-sheet structure with, for amide I, a maximum at 1634 cm^−1^ and a marked shoulder around 1690 cm^−1^ together with amide II at 1536 cm^−1^. These spectral features are typical of β-sheet structures (Arrondo et al. 1993; Goormaghtigh et al. 1994b; Barth 2007). The picture is less clear for the amino acid side chains. We compare below the position of amino acid contributions that appear in Fig. 1 with the values reported for free amino acids reported by Wolpert and Hellwig (2006). In the amide I region, arginine side chain contribution is rather correctly obtained with bands at 1673 and 1646 cm^−1^ (1673 and 1633 cm^−1^ for the free amino acid) though the relative absorbance of the two contributions is not respected. Lysine presents a maximum at 1640 cm^−1^ (1636 cm^−1^ for the free amino acid). Asparagine and glutamine profiles do not match the two bands expected near 1672–1681 and 1610–1618 cm^−1^ found in free amino acids. In the amide II region, aspartate presents a maximum at 1580 cm^−1^ and glutamate at 1572 cm^−1^. These values are significantly higher than the 1570 and 1559 cm^−1^ reported for free amino acids. Upon subtraction of amino acid side chain contribution (see later) it appeared that the specific contribution of glutamate had to be shifted from 1559 to 1570 cm^−1^ to avoid negative absorbance values after subtraction. This correction was confirmed by the observation glutamate ν_as_(COO^−^) absorbs at 1572 in Fig. 1 and was applied in this work. Tyrosine contribution maximum appears at 1514 cm^−1^ in Fig. 1 while it is found at 1518 cm^−1^ in free amino acids. In the course of this work, we also observed that the band had to be shifted to 1514 cm^−1^ to account to the observed position in protein spectra. This is the second modification we brought to the parameters describing the side chain contributions in this paper. This latter modification was further confirmed by direct observation of the position of the tyrosine ring vibrations on the second derivatives of the original spectra. As this band is very narrow, it is strongly enhanced by taking the second derivative and thereby easily observable as shown in the inset of Fig. 1a. The inset shows clearly that 1518 cm^−1^ does not match the observation while 1514 cm^−1^ does.

### Subtraction of amino acid side chains

**Fig. 2:**
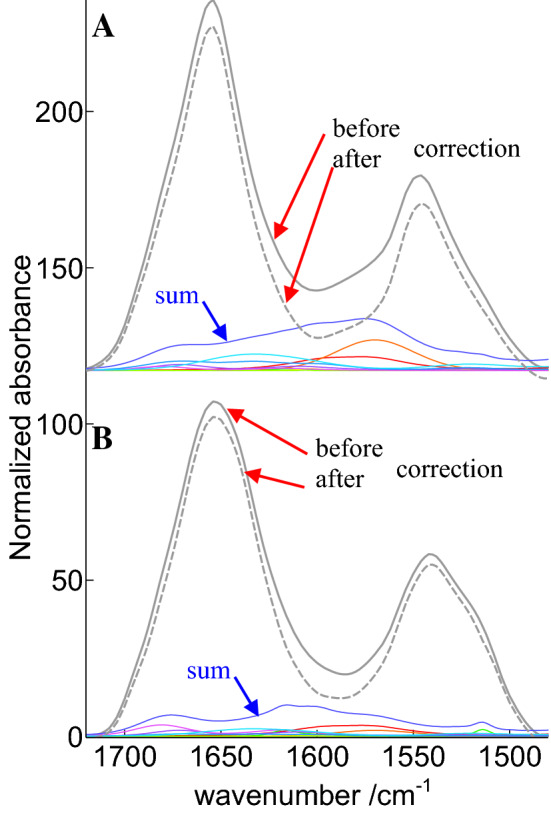
Contribution and subtraction of amino acid side chain contributions for **a** Alpha-2-MRAP and **b** Elafin. The gray lines show the recorded spectra (plain line) and the corrected spectra (dashed line). The corrected spectra are obtained after subtraction of the sum of the amino acid contributions (blue line). The individual contributions of the side chains are shown in color, the blue line is the sum of these individual contributions. Absorbance is in arbitrary units and spectra have been offset for the clarity of the figure

Taken together, the overall contribution of side chains in the amide I–amide II spectral range may vary by a factor larger than 2. To obtain a general overview of potential differences in the relative contributions of side chain absorption for each protein, the fractional content of each amino acid was multiplied by the intensity of its contribution in the amide I–amide II spectral range. Proteins were then sorted according to the sum of these contributions. Such an approach indicates that side chains in alpha-2-MRAP (PDB code 2P03) absorb more than twice as much as in Elafin (PDB code 1FLE), as illustrated in Fig. 2.

**Fig. 3:**
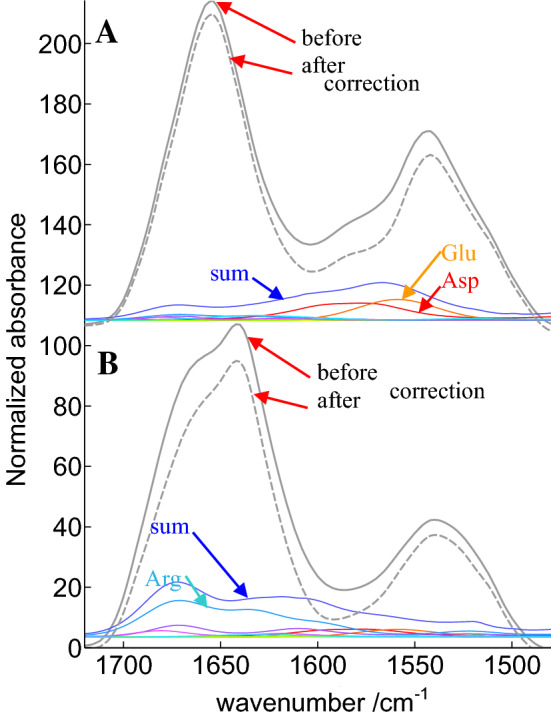
Illustration of the subtraction of amino acid side chain contributions for **a** calmodulin and **b** endo-1,4-beta-xylanase. The gray lines show the recorded spectra (plain line) and the corrected spectra (dashed line). The corrected spectra are obtained after subtraction of the sum of the amino acid contributions (blue line). The individual contributions of the side chains are shown in color. The contribution of glutamic and aspartic acids is identified in **a** for calmodulin and of arginine side chains in **b** for endo-1,4-beta-xylanase. Absorbance is in arbitrary units and spectra have been offset for the clarity of the figure

While the global area assigned to amino acid side chains is quite different in Fig. 2a, b, in both cases, the amino acid contribution appears as a rather broad baseline underlying amide I and amide II. Yet, depending on amino acid composition, this overall contribution can be definitively more intense in a specific spectral region. Some proteins have indeed “anomalous” content in some specific amino acids. In the amide I spectral range, arginine is one of the most problematic as it presents a strong contribution at 1673 and 1633 cm^−1^. The mean content in arginine in cSP92 is 4.3% but some protein have none such as metallothionein (PDB code 4MT2) while arginine represents 14% of all amino acids in cathepsin G (PDB code 1CGH) as reported (De Meutter and Goormaghtigh 2020). Furthermore, asparagine also represents 4.5% of Cathepsin G amino acid content, which is average, but adds up to arginine contribution in the same spectral region. It must be noted that some specific protein/peptides such as the antibacterial peptide PR-39 contains almost 30% arginine which dominates the FTIR spectrum the amide–I amide II region (Cabiaux et al. 1994). In the amide II spectral range, ionized carboxylic acids bring a large contribution. Carboxylic acids, i.e., the sum of aspartic and glutamic acids, represent 11.3% of cSP92 proteins amino acids but in calmodulin (1PRW) they represent 22.6% and only 3.94% in endo-1,4-beta-xylanase (2JIC). The extreme values reported above for endo-1,4-beta-xylanase and calmodulin suggest the shape of amide I and II could be significantly affected. Figure 3 reports for both proteins the recorded spectra and the corrected spectra, along with the different amino acid side chains contributions and their sum.

It can be observed in Fig. 3 that, for endo-1,4-beta-xylanase, arginine side chains bring a very significant contribution in particular near 1673 cm^−1^. The shape of its spectrum is dramatically changed upon subtraction of side chain contributions. Carboxylic acids that are prominent in calmodulin rather affect the shape of amide II, which is also rich in information on protein secondary structure (Goormaghtigh et al. 2006). It is, therefore, legitimate to question the effect of amino acid side chain contributions on the accuracy of secondary structure prediction based on FTIR spectra.

**Fig. 4:**
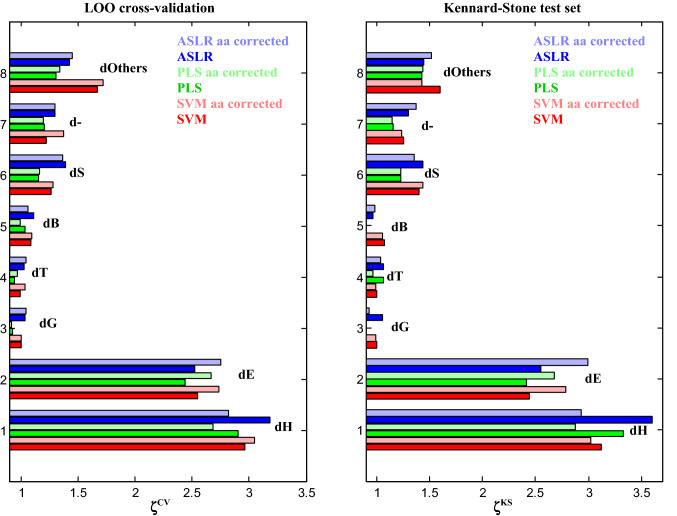
ζ values for validation of the 8 DSSP-defined secondary structure element and “Others”, explained in the text. The left panel reports results for a leave-one-out cross-validation and the right panel the Kennard–Stone test set (1/3 of the protein spectra). For each structure, 3 prediction methods have been used, ASLR, PLS and SVM. For each method, raw spectra or spectra corrected for amino acid side chain contributions have been used. The color code is indicated in each of the panels. For PLS, the number of LVs has been determined as described on Fig. S3

Methods used to relate spectral shape to secondary content are numerous. As they rely on different principles, they could be more or less prone to errors related to side chain absorption. It is therefore important to test more than one. Here, three different approaches will be tested. A simple linear regression using a small number of absorbance values, usually the absorbance at 2–5 wavenumbers, is the ascending stepwise regression (ASLR) which adds, step by step, the best absorbance in the linear model (Goormaghtigh et al. 2006, 2009). Partial least square regression (PLS) is probably the most popular multivariate approach in chemometrics, including in the field of FTIR spectroscopy of proteins (Navea et al. 2005; Wang et al. 2008). It is well designed to deal with co-linearity. As non-linear models could shed a different light on the predictions, we also included a support vector machine (SVM) modified for solving regression problems (Ghorbani et al. 2016). The error of prediction was evaluated by both a leave-one-out (LOO) cross-validation and a single protein test set made out of one third of the protein spectral database. In the latter case, proteins were selected by the Kennard–Stone algorithm (Kennard and Stone 1969) (see Methods). For both approaches, proteins tested are never part of the training set. Results appear in Fig. 4 for all DSSP-defined structures. Details of the data presented in Fig. 4 are reported in Table S2.

Considering the cross-validation results (Fig. 4, left panel), it immediately appears that the FTIR spectra bring little information for the prediction of the content in the minor structures such as dG, dT and dB. The bright colors refer to the prediction obtained from the raw spectra, the pastel colors to the prediction obtained from spectra corrected for amino acid side chain contributions. For the α-helix (dH), there is a slight improvement only for SVM prediction. For the β-sheet (dE), there is a systematic improvement for all three prediction methods. Using SVM, ζ^cv^ for dE increases from 2.55 to 2.73 while the RMSECV is decreased from 5.37 to 5.02% (see Table S2 for detailed figures). Using ASLR, the RMSECV for dE decreases from 5.42 to 4.97, which is significant. Prediction of the “Others” structure also gains in accuracy for all methods when side chain contributions are removed. The effect is less marked for d −. The picture is less clear for the other minor structures which are characterized anyway by ζ^CV^ values close to 1. When looking at the right panel of Fig. 4, the Kennard–Stone test set obtains predictions with higher values for ζ^KS^ as compared to ζ^CV^. It is important to stress that this effect is not due not to a decrease in the error of prediction but to the larger standard deviation of the secondary structure content in the test set, obviously related to the criterion applied for the selection of the test set (see Table S2). Again, subtracting the amino acid side chain contributions clearly benefits β-sheet content prediction but not the other structures. In particular, the correction degrades α-helix content prediction. Proteins for which the best improvement of dE prediction was observed after side chain subtraction were scrutinized for particular amino acid content but no general rule could be obtained (data not shown).

The DSSP analysis of high-resolution structure described in the PDB is not unique. A series of structure definitions has been defined over the years and can result in quite different secondary structure content. The extent of secondary structure variation that can result from using different definitions has been described for several methods for cSP92 in De Meutter and Goormaghtigh (2021c). It was of interest to test another approach that provides a more detailed analysis. XTLSSTR (see Methods), which is significantly different from DSSP, has been selected. The same analysis was therefore performed after obtaining the secondary structures according to XTLSSTR instead of DSSP. Even though the secondary structure content may be very significantly different from those obtained by DSSP, the results (Fig. S4) are similar: significant improvement for the prediction of β-sheet content but not for the other structures. One interest of XTLSSTR is that it makes the difference between the core of the structure (“H” and “E” for the α-helix and b-strand, respectively) and for part of it that are present on the side or are not connected, indicated by “h” and “e”, respectively. It can be observed on Figure S4 that “H” and “H + h”, i.e., the total α-helix content, are better predicted before side chain subtraction while “E”, “e” and “E + e” are much better predicted after subtraction of the side chain contribution. Interestingly, prediction for xE (RMSECV = 4.86) is significantly better than the prediction of the total (xe + xE) β-strand content (RMSECV = 5.97). A more detailed analysis is reported on Figure S5 for the β-sheet structure. The few proteins whose prediction has been improved upon subtraction of the side chain contribution are singled out. Yet, when analyzing the amino acid content of these proteins, there is no obvious explanation for the improvement (Fig. S5). This could be due to the fact the model built is global and the proteins that have apparently a bad prediction may not be the cause of the loss of prediction capacity but the result of the adjustment of the model to account for anomalous behavior found in other proteins.

The results presented above confirm that dE prediction is improved by subtraction of side chain contributions but raise the question of the rationale for this subtraction in general. One of the possible reasons why side chain contribution might not interfere as much as expected for all structures is there exists a degree of correlation between secondary structure content and amino acid composition.

### Impact of the correlation between amino acid composition and secondary structure

There is a well-documented prevalence of certain amino acid for specific secondary structures. This has been observed since the 1970s in the pioneer work of Chou and Fasman (1974) or Garnier (1978), on large survey of PDB proteins (Otaki et al. 2010) as well as among cSP92 protein set (De Meutter and Goormaghtigh 2020). In cSP92, correlation coefficients between secondary structure content and each amino acid content can reach 0.4–0.5 (Fig. S1). This implicates that a fraction of the amino acid side chain contribution to the spectrum is intimately linked to secondary structure content. In such a case, the effect of subtraction is expected to be neutral. It is interesting to apply a method such as ASLR to determine the secondary structure content from amino acid content. The “spectrum” to be analyzed is now the content in the 20 amino acid. Such an attempt appears on Figure S2 for the DSSP-defined structures. The best prediction is found for the α-helix content that can be expressed as 39.1 + 3.3*[Leu] − 4.8*[Pro]—1.9*[Thr]  − 2.3*[Val] + 1.3*[Ala] (Fig. S2) with a RMSECV of 12.2% from a standard deviation of 18.5%, i.e., ζ = 1.50. Though to a less extent for the other secondary structures, there is definitively a prediction potential. ASLR reveals the best amino acids for this prediction reported in the equation above for dH. It is important to note here that these amino acids do not contribute much in the amide I—amide II spectral range. In general, except for Glu, the amino acid that contributes most in the amide I–amide II region of the spectrum have weak correlation with α-helix, β-sheet and “Others” content (Fig. S1). When the computation was repeated with only the first 8 amino acids which most contribute to the FTIR spectrum in the amide range (Glu, Asp, Arg, Lys, Gln, Asn, Tyr, His), none of the structure could be predicted with ζ > 1.08, i.e., a correlation coefficient of 0.33 at best (not shown). It can be concluded that co-linearity is not an important factor responsible for the relative failure of side chain subtraction to improve significantly secondary structure prediction.

Overall, we have shown that the β-sheet content prediction is improved upon subtraction of amino acid side chain contributions in the amide I–amide II spectral range. Improvement is relatively important, for instance RMSECV decreases from 5.42 to 4.97% when ASLR is used. In fact, all methods, PLS, SVM and ASLR reach the same conclusion. The other structures do not significantly benefit from side chain subtraction, in some cases prediction is even degraded. We showed that co-linearity between secondary structure content and amino acid composition is not a main limitation for improving secondary structure prediction. We also showed that, even though based on different definitions, using secondary structures defined by DSSP and XTLSSTR drives to the same conclusion: only the β-sheet content prediction clearly benefits from side chain subtraction. It must be concluded that the very rough description of side chain absorbance which does not take into account their large variations related to their environment limits the potential to improve all secondary structure predictions.

## Supplementary Information

Below is the link to the electronic supplementary material.

Supplementary file1 (PDF 1691 KB)

## Data Availability

A Matlab code for reconstructing side chain contributions is provided in Supplementary materials along with the parameters used.

## Availability of data and material

Materials is available on request from the authors.
