# Supplementary material for: Amino acid side chain contribution to protein FTIR spectra: impact on secondary structure evaluation
Source: Eur Biophys J. 2021 Feb 8;50(3-4):641–51. doi: 10.1007/s00249-021-01507-7 (PMC8189991; doi:10.1007/s00249-021-01507-7)
Supplement: Supplementary file 1 — Supplementary file1 (PDF 1691 KB) [file 249_2021_1507_MOESM1_ESM.pdf]

Supplementary materials for

Amino acid side chain contribution to protein FTIR spectra: impact on secondary structure evaluation

Joëlle De Meutter and Erik Goormaghtigh

Center for Structural Biology and Bioinformatics, Laboratory for the Structure and Function of Biological Membranes, Campus Plaine CP206/02; Université Libre de Bruxelles, B1050 Brussels, Belgium

Table of Contents

Figure S1: Correlation between amino acid content and secondary structure 2
Figure S2: Prediction of secondary structure content from amino acid concentration 3
Figure S3: Determination of the number of required LVs for PLS.. 4
Figure S4: ASLR prediction of XTLSSTR-defined structures before and after side chain contribution subtraction 5
Figure S5: ASLR prediction of XTLSSTR-defined structures: detailed analysis of β-strand 7
Table S1: Parameters describing side chain contribution between 1800 and 1400 cm^{-1}. 8
Table S2: SVM, PLS and ASLR secondary structure prediction results 9

Figure S1: Correlation between amino acid content and secondary structure

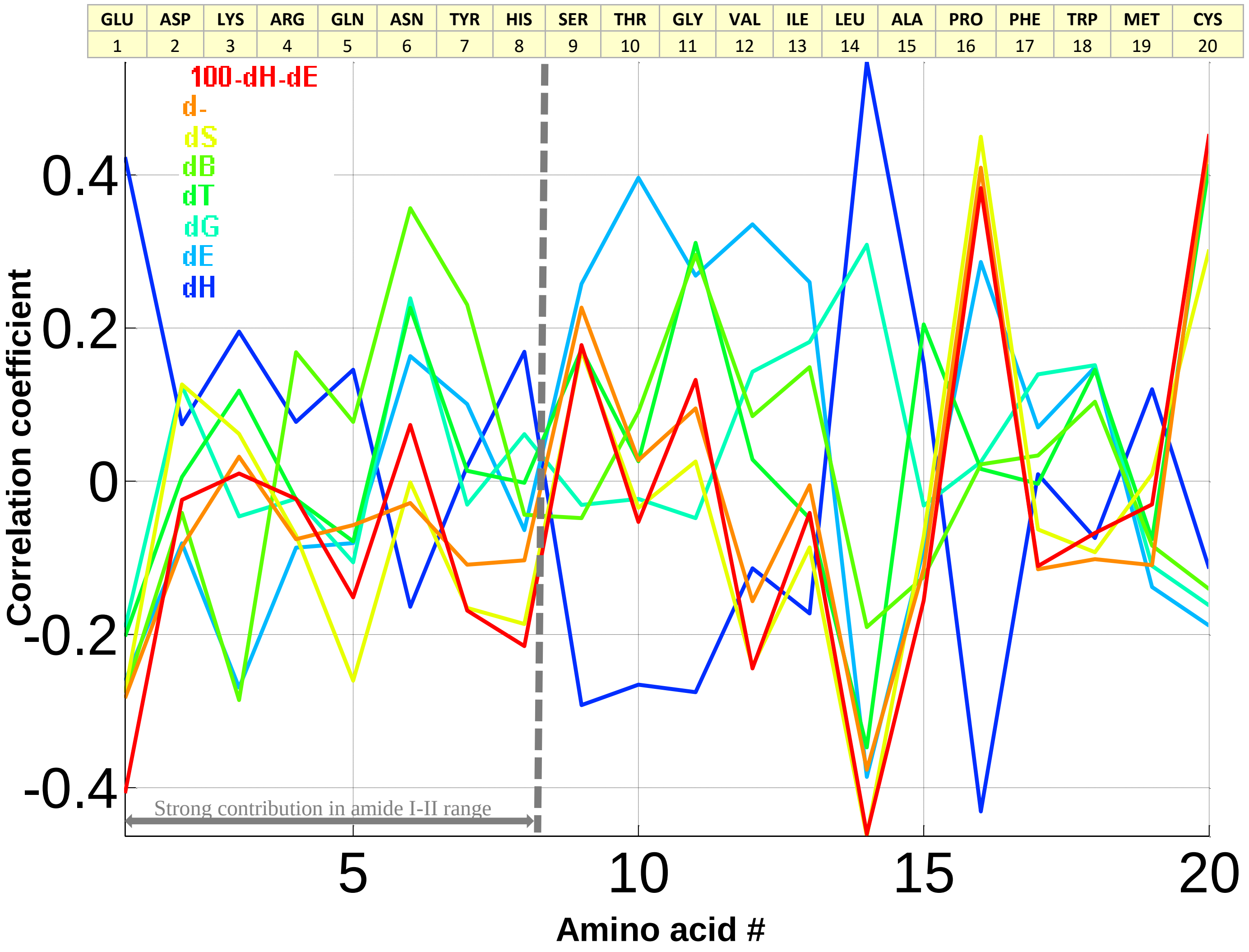

Figure S1: correlation coefficient in cSP92 protein spectra between the content in 8 structures defined by DSSP and the content in the 20 amino acids. The color code for the 8 structures is indicated in the upper left corner and the amino acids appear on top of the figure. The amino acids present on the left of the vertical dotted line are those whose contribution to the amide I – amide II region of the spectrum is more significant.

Figure S2: Prediction of secondary structure content from amino acid concentration

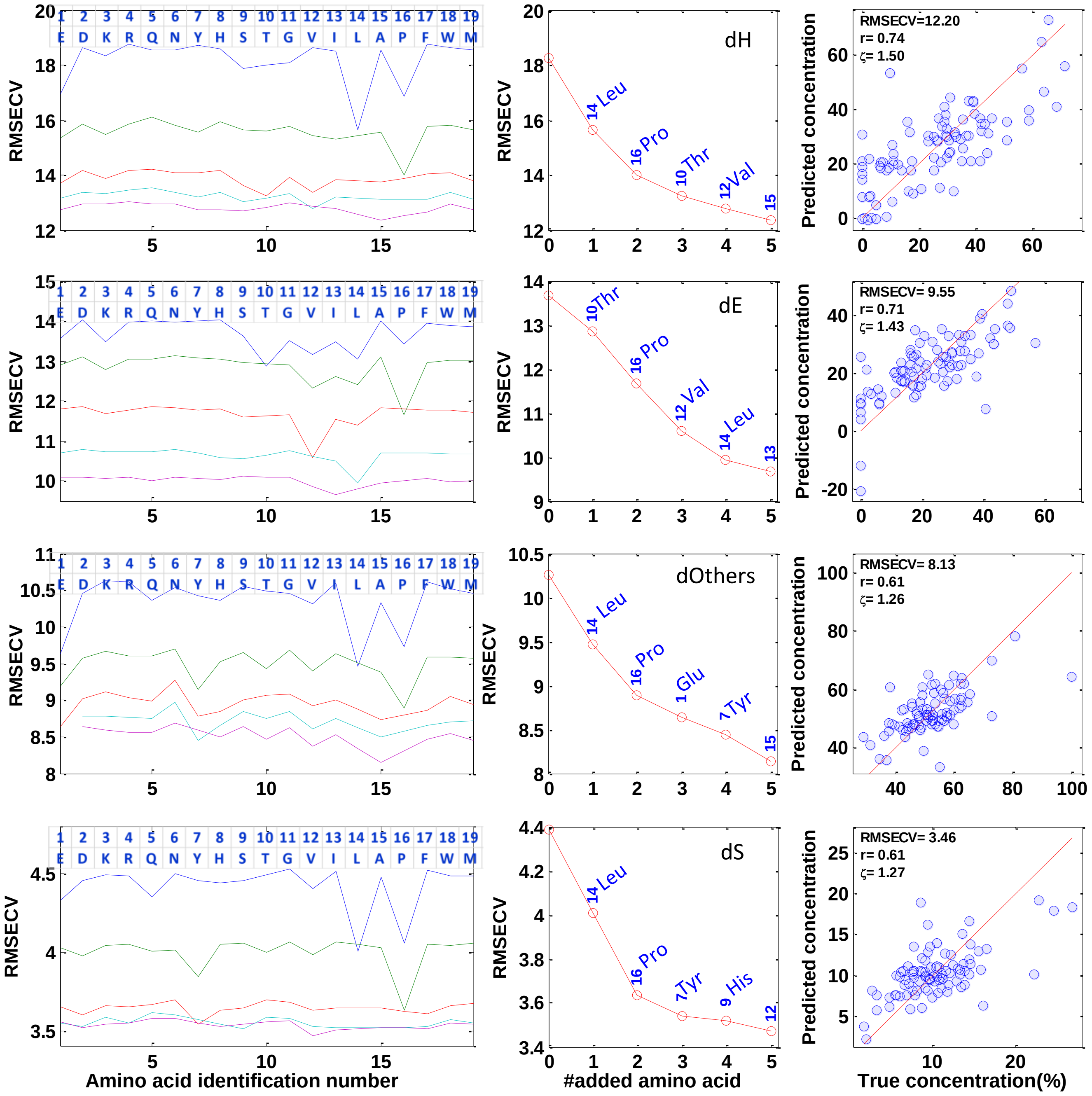

Figure S2. Prediction of dH, dE, dOthers and dS based on amino acid content. Structure dT, dB and dG had ζ values below 1.25 and are not reported here. The left column reports the drop in RMSECV (%) as the one (blue line), two (green line), three (red line) etc. amino acid concentrations (%) are added to the model. The middle column summarized the drop in RMSECV as a function of the number of added amino acid concentrations and the right column reports the predicted concentration for each structure versus the "true" concentration.

For instance, the α-helix (dH) content can be computed as 39.1 + 3.3*[Leu] - 4.8*[Pro] - 1.9*[Thr] - 2.3*[Val] + 1.3*[Ala] with a RMSECV of 12.2% from a standard deviation of 18.5%. Because cysteine is a source of large error, its contribution is not presented. The very high error brought by cysteine is due to the fact more than half of the proteins have no cysteine and 80 % of them have less than 1%.

Figure S3: Determination of the number of required LVs for PLS

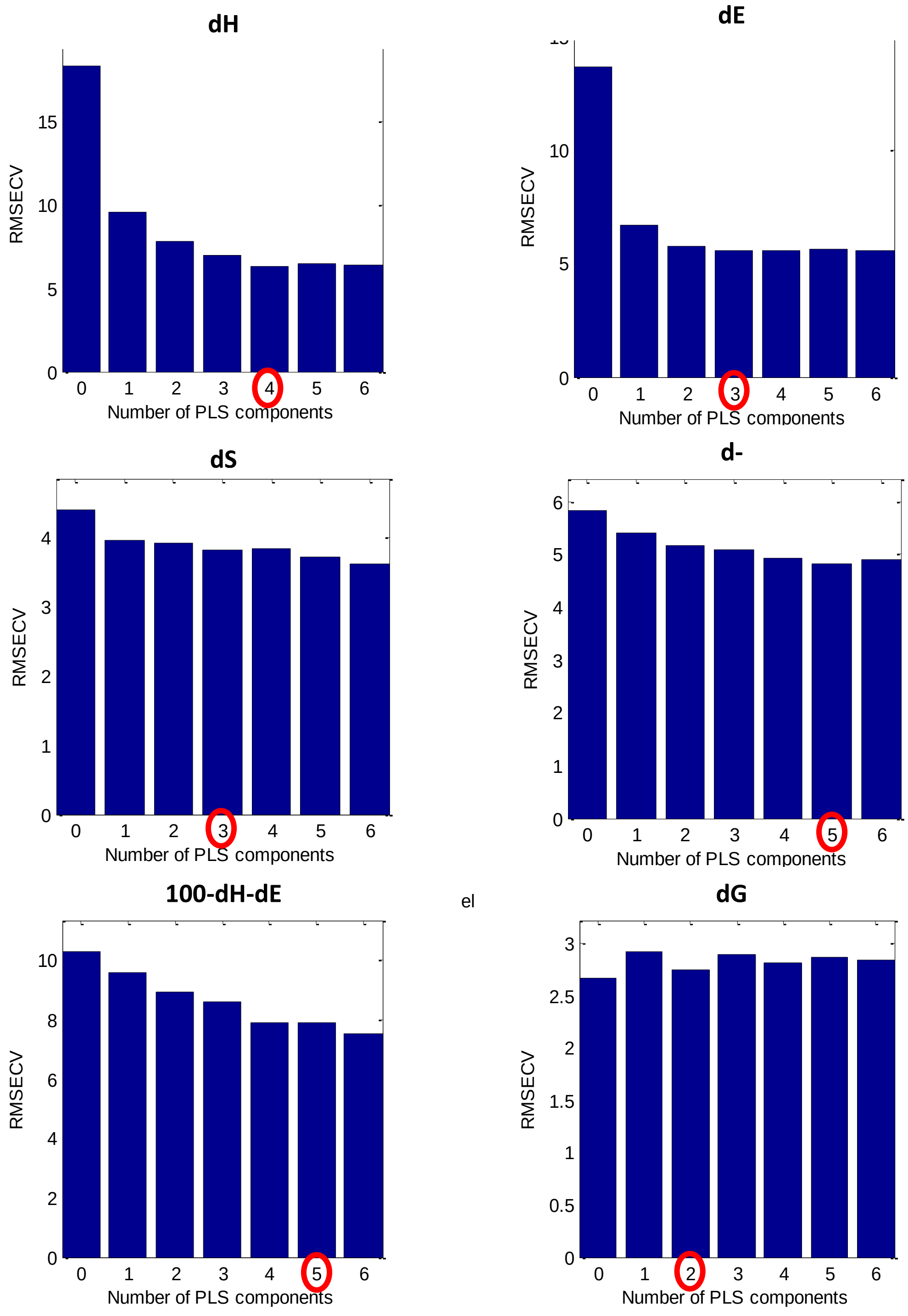

Figure S3. Evolution of RMSECV as a function of the number of latent variables (LVs) in PLS models. The numbers circled in red have been used in this work. Prediction appears not significant for dG. The same result is obtained for dB, dT and dI (not shown). These results were obtained on the original spectra.

Figure S4: ASLR prediction of XTLSSTR-defined structures before and after side chain contribution subtraction

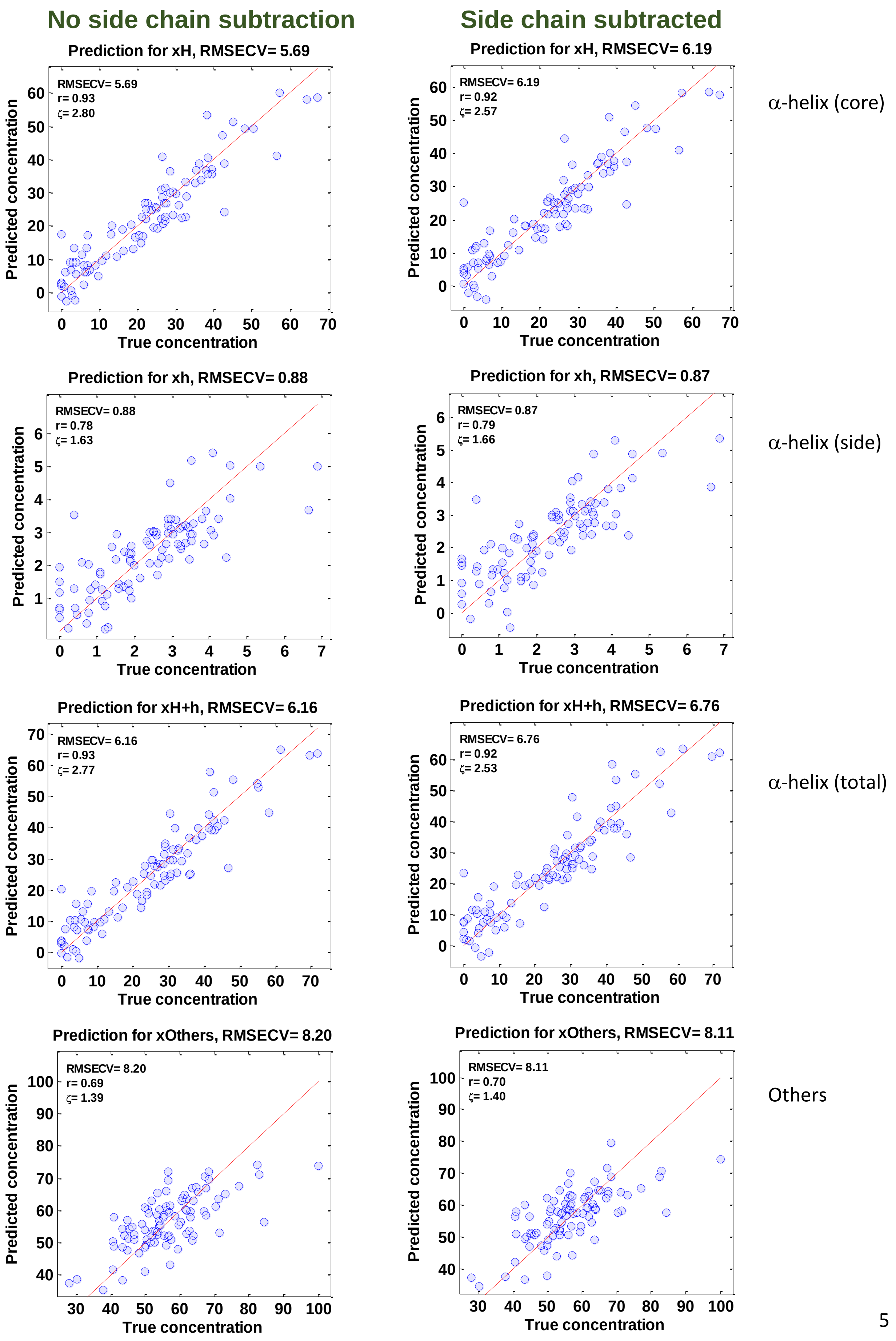

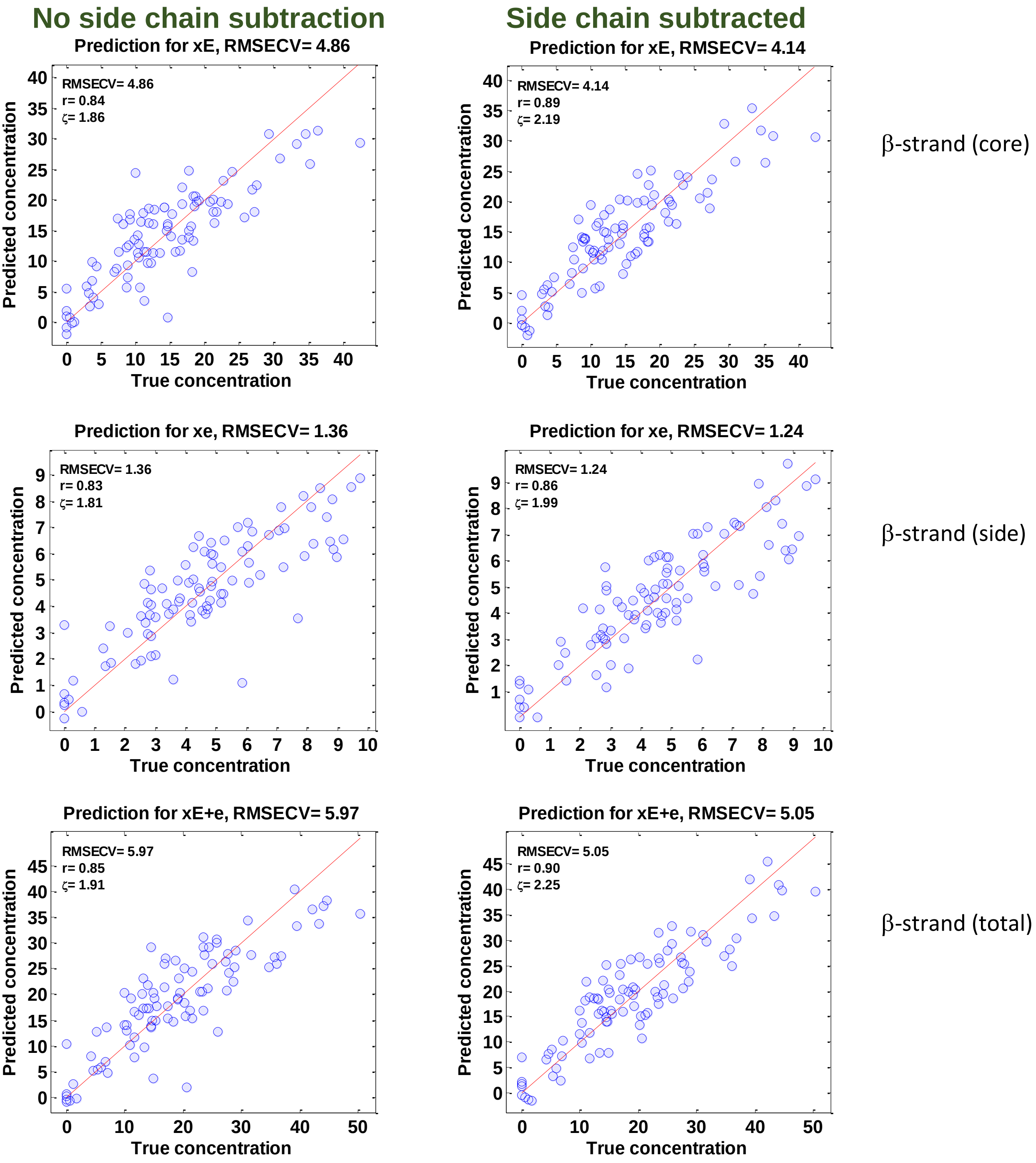

Figure S4: Relation between the actual and predicted secondary structure content. The actual structure content is obtained from the analysis of the high resolution structure by the definitions designed by XTLSSTR (King and Johnson 1999). XTLSSTR is an alternative to DSSP to obtain secondary structure content from PDB files. It defines α–helix (H), β-strand (E), and a series of structures that are not abundant enough to obtain good prediction, including 3_{10}-helix, hydrogen-bonded turn, non-hydrogen-bonded turn and poly(L-proline) II type 3_1-helix. Lower case letters indicate residues that are not part of the core of the main structures, but are located either at the end of a structure or disconnected from it. Prediction have been obtained in LOO cross-validation by ASLR using 4 wavenumbers. Evaluation was carried out before (left column) and after (right column) subtraction of the side chain contributions.

Reference

King SM, Johnson WC (1999) Assigning secondary structure from protein coordinate data. Proteins Struct Funct Genet 35:313–320. https://doi.org/10.1002/(SICI)1097-0134(19990515)35:3

Figure S5: ASLR prediction of XTLSSTR-defined structures: detailed analysis for the β-strand

No side chain subtraction

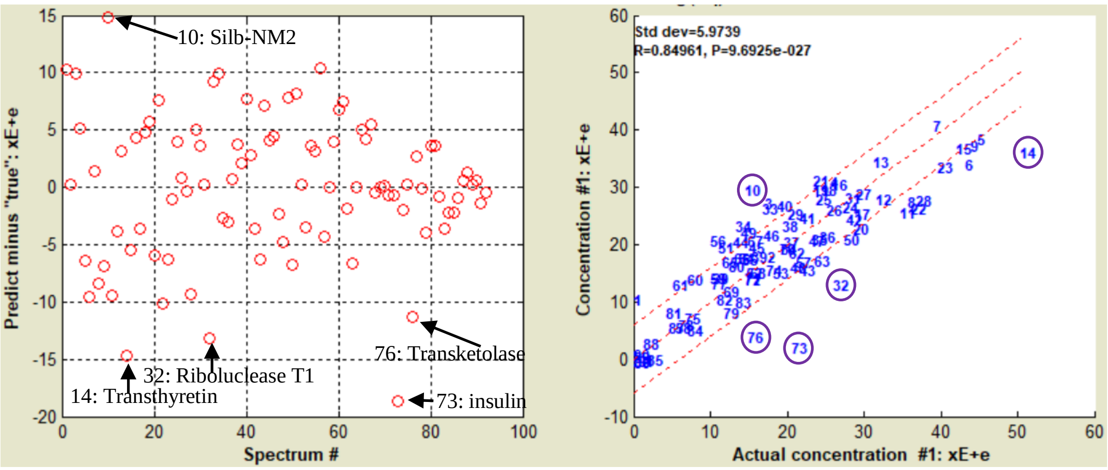

Side chain subtracted

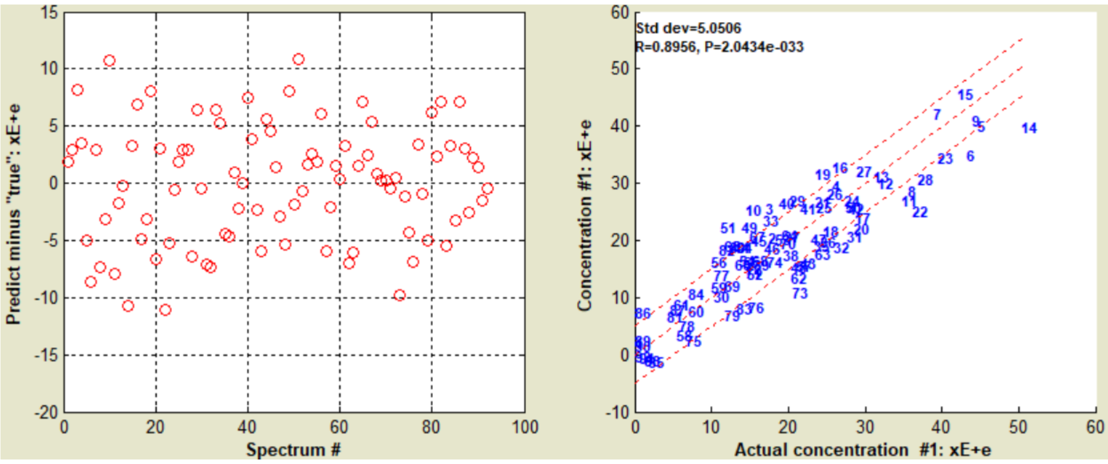

Figure S5: Detailed analysis of the prediction of the total β-strand content defined by XTLSSTR by ASLR before and after subtraction of the side chain contribution. Left: difference between predicted and actual content as a function of the spectrum number, right: the predicted concentrations are reported as a function of the actual concentrations. The best fit is indicted by the central dashed line, the two other red lines indicate ± one standard deviation. The amino acid composition is reported below for these proteins.

	GLU	ASP	LYS	ARG	GLN	ASN	TYR	HIS
10: Silb-NM2	7.78	5	4.44	6.67	4.44	1.67	1.67	1.11
14: Transthyretin	9.45	3.94	6.3	3.15	0	2.36	3.94	3.15
32: Riboluclease T1	5.77	5.77	0.96	0.96	2.88	8.65	8.65	2.88
73: Insulin	7.84	0	1.96	1.96	5.88	5.88	7.84	3.92
76: Transketolase	7.25	5.29	5.29	4.23	3.47	3.32	3.47	2.72
mean value in cSP92	5.75	5.55	6.12	4.31	3.70	4.56	3.40	2.36

Amino acid composition of the proteins singled out in the figure above.

Table S1: Parameters describing side chain contribution between 1800 and 1400 cm^{-1}.

The parameters below are presented in a format that can be read immediately by MatLab. A copy/paste of the lines below should load the data. A plot is finally provided, using the relative amino acid mean content found in cSP92.

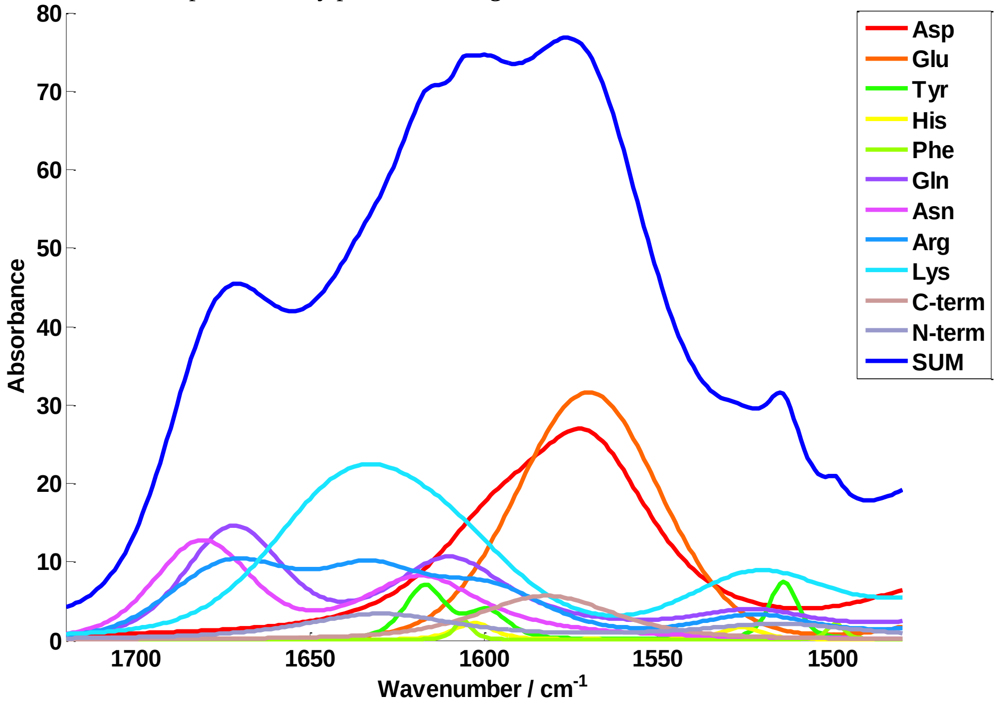

Plot of the side chain contribution using for each amino acid the parameters listed below. The mean concentration found in cSP92 has been used for each of them.

```
%Parameters describing the absorption of different side chains
%function SideChains

ic=1; %#1
cust.sidechain(ic).color=[1 0 0];
cust.sidechain(ic).shortname='Asp';
cust.sidechain(ic).naa=5.64;  %fraction of residues in the protein (here median content in % in cSP92)
cust.sidechain(ic).npH=2;   %number of pK in the residue
cust.sidechain(ic).pK=4.25;  %pKa
cust.sidechain(ic).nc=[5 3;  1 1];    % number of components (H-high pH, H-low pH; D-high pD, D-low pD)
cust.sidechain(ic).freq=[1598 1570 1472 1421 1395 1729 1456 1410 1584 1713]; %H-high pH, H-low pH, D-high pD, D-low pD
cust.sidechain(ic).FWHH=[44 44 44 44 44 50  44 44 44 44];
cust.sidechain(ic).ODmax=[349/2 402 70 186 351 282 71 131 820 290]; %349/2 because shoulder
cust.sidechain(ic).fg=[.3 .3 .3 .3 .3 .6 .8 .8 .8 .8];

ic=ic+1; %#2
cust.sidechain(ic).color=[1 .4 0];
cust.sidechain(ic).shortname='Glu';
cust.sidechain(ic).naa=5.78;  %fraction of residues in the protein (here median content in % in cSP92)
cust.sidechain(ic).npH=2;   %number of pH forms
cust.sidechain(ic).pK=3.65;  %pKa
cust.sidechain(ic).nc=[3 3;  1 1];    % number of components (H-high pH, H-low pH; D-high pD, D-low pD)
cust.sidechain(ic).freq=[1570 1451 1404 1728 1454 1417 1567 1706]; % Glu 1570, own observation
cust.sidechain(ic).FWHH=[48 48 48 56 56 56 34 45];
cust.sidechain(ic).ODmax=[546 63 290 219 23 33 830 280];
cust.sidechain(ic).fg=[.9 .9 .9 .5 .5 .5 .4 .4];

ic=ic+1; %#3
cust.sidechain(ic).color=[.15 1 0];
cust.sidechain(ic).shortname='Tyr';
cust.sidechain(ic).naa=3.08;  %fraction of residues in the protein  (here median content in % in cSP92)
cust.sidechain(ic).npH=2;   %number of pH forms
```

```
cust.sidechain(ic).pK=10;  %pKa
cust.sidechain(ic).nc=[4 4;2 2];
cust.sidechain(ic).freq=[1601 1560 1499 1444 1617 1599 1514 1455 1603 1500 1615 1516]; %1514 instead 1518, own observation
cust.sidechain(ic).FWHH=[14 14 14 14 14 14 10 14 14 14 9 7];   %10 taken from Rahmelow
cust.sidechain(ic).ODmax=[320 319 468 63 222 115 241 104 350 650 160 500];
cust.sidechain(ic).fg=[.5 .5 .5 .5 .5 .5 .5 .5 .5  .5 0 .4 0 .4];

ic=ic+1; %#4
cust.sidechain(ic).color=[1 1 0];
cust.sidechain(ic).shortname='His';
cust.sidechain(ic).naa=2.31;  %fraction of residues in the protein (here median content in % in cSP92)
cust.sidechain(ic).npH=2;   %number of pH forms parameters are shown
cust.sidechain(ic).pK=8.97;  %pKa
cust.sidechain(ic).nc=[5 3; 1 1];
cust.sidechain(ic).freq=[1591 1568 1498 1465 1439 1603 1526 1438 1596 1596];
cust.sidechain(ic).FWHH=[14 14 14 14 14 14 14 14 14 14];
cust.sidechain(ic).ODmax=[10 97 74 10 30 97 74 30 70 70]; %high pH intensity - low pH intensities
cust.sidechain(ic).fg=[.4 .4 .4 .4 .4 .4 .4 .4 .4 .4];

ic=ic+1; %#5
cust.sidechain(ic).color=[.6 1 0];
cust.sidechain(ic).shortname='Phe';
cust.sidechain(ic).naa=3.95;  %fraction of residues in the protein (here median content in % in cSP92)
cust.sidechain(ic).npH=1;   %number of pH forms
cust.sidechain(ic).nc=[4; 1];    % number of components
cust.sidechain(ic).freq=[1606 1499 1457 1446 1494];
cust.sidechain(ic).FWHH=[6 6 6 6 6];
cust.sidechain(ic).ODmax=[66 55 35 20 80];
cust.sidechain(ic).fg=[.2 .2 .2 .2 .2];

ic=ic+1; %#6
cust.sidechain(ic).color=[.6 .3 1];
cust.sidechain(ic).shortname='Gln';
cust.sidechain(ic).naa=3.71;  %fraction of residues in the protein (here median content in % in cSP92)
cust.sidechain(ic).npH=1;   %number of pH forms
cust.sidechain(ic).nc=[5; 1];
cust.sidechain(ic).freq=[1672 1610 1523 1452 1411 1635];
cust.sidechain(ic).FWHH=[32 44 44 44 44 36];
cust.sidechain(ic).ODmax=[360 275 79 72 149 560];
cust.sidechain(ic).fg=[.8 0 0 0 0 .6];

ic=ic+1; %#7
cust.sidechain(ic).color=[.9 .3 1];
cust.sidechain(ic).shortname='Asn';
cust.sidechain(ic).naa=4.34;  %fraction of residues in the protein (here median content in % in cSP92)
cust.sidechain(ic).npH=1;   %number of pH forms
cust.sidechain(ic).nc=[2; 1];
cust.sidechain(ic).freq=[1681 1618 1502 1421 1404 1648];
cust.sidechain(ic).FWHH=[32 44 44 44 4431];
cust.sidechain(ic).ODmax=[274 187 56 99 103 570];
cust.sidechain(ic).fg=[.8 0 0 0 0 .6];

ic=ic+1; %#8
cust.sidechain(ic).color=[.1 .6 1];
cust.sidechain(ic).shortname='Arg';
cust.sidechain(ic).naa=3.83;  %fraction of residues in the protein  (here median content in % in cSP92)
cust.sidechain(ic).npH=1;   %number of pH forms
cust.sidechain(ic).nc=[6; 2];
cust.sidechain(ic).freq=[1673 1633 1598 1522 1475 1454 1608 1586];
cust.sidechain(ic).FWHH=[40 40 40 40 40 40 40 21 22];
cust.sidechain(ic).ODmax=[235 219 151 75 18 28 500 460];
cust.sidechain(ic).fg=[.9 .5 .5 .5 .5 .5 .5 .4 .4];

ic=ic+1; %#9
cust.sidechain(ic).color=[.1 .9 1];
cust.sidechain(ic).shortname='Lys';
cust.sidechain(ic).naa=5.84;  %fraction of residues in the protein (here median content in % in cSP92)
```

```
cust.sidechain(ic).npH=1;   %number of pH forms
cust.sidechain(ic).nc=[7; 0];    % number of components
cust.sidechain(ic).freq=[1651 1634 1608 1521 1476 1462 1445];
cust.sidechain(ic).FWHH=[46 48 48 48 48 48 48];
cust.sidechain(ic).ODmax=[108 242 152 138 36 35 28];
cust.sidechain(ic).fg=[.5 .7 .7 .7 .7 .7 .7];

ic=ic+1; %#10
cust.sidechain(ic).color=[.8 .6 .6];
cust.sidechain(ic).shortname='C-term';
cust.sidechain(ic).naa=1;  %fraction of residues in the protein (here median content in % in cSP92)
cust.sidechain(ic).npH=2;   %number of pH forms
cust.sidechain(ic).pK=5.5;  %pKa
cust.sidechain(ic).nc=[1 1; 1 1];
cust.sidechain(ic).freq=[1582 1740 1592 1720]; %from Rahmelow
cust.sidechain(ic).FWHH=[47 50 32 45];
cust.sidechain(ic).ODmax=[575 170 830 230];
cust.sidechain(ic).fg=[.4 1 .4 .8];

ic=ic+1; %#11
cust.sidechain(ic).color=[.6 .6 .8];
cust.sidechain(ic).shortname='N-term';
cust.sidechain(ic).naa=1;  %fraction of residues in the protein (here median content in % in cSP92)
cust.sidechain(ic).npH=2;   %number of pH forms
cust.sidechain(ic).pK=9.5;  %pKa
cust.sidechain(ic).nc=[1 2; 0 0];
cust.sidechain(ic).freq=[1560 1630 1515]; %from Rahmelow
cust.sidechain(ic).FWHH=[46 54 60];
cust.sidechain(ic).ODmax=[450 330 200];
cust.sidechain(ic).fg=[0 .2 0];



%The following lines compute and draw side chain contributions defined by the parameters above
pH=7.4;  %pH of the experiment
naatot=100; %total number of amino acid in the protein, here 100 because side chain content expressed in %
xmax=1720;
xmin=1480;
np=xmax-xmin+1;
xcm=linspace(xmax,xmin,np);
spres=0;
SideChain.spind=[];


%Tabulation of the values in convenient variables
ncontrib=length(cust.sidechain);  %number of side chains
for k=1:ncontrib
  naa(k)=cust.sidechain(k).naa;
  perD(k)=0; %percent of deuterated side chains
  npH(k)=cust.sidechain(k).npH;
  if npH(k)>1
    pK(k)=cust.sidechain(k).pK;
  end
  index=0;
  for l=1:2 %boucle sur formes H/D
    for m=1:npH(k) %boucle sur chaque forme ionisée
      nc(k,l,m)=cust.sidechain(k).nc(l,m);
      for n=1:nc(k,l,m)
        index=index+1;
        freq(k,l,m,n)=cust.sidechain(k).freq(index);
        FWHH(k,l,m,n)=cust.sidechain(k).FWHH(index);
        ODmax(k,l,m,n)=cust.sidechain(k).ODmax(index);
        fg(k,l,m,n)=cust.sidechain(k).fg(index);
      end
    end
  end
end
```

```
167
168 % Fraction of low and high pH form
169 for k=1:ncontrib
170   perpHlow(k)=100;
171   if npH(k) >= 2
172     perpHlow(k)=10^(-pH)/10^(-pK(k));
173     perpHlow(k)=100*perpHlow(k)/(1+perpHlow(k));
174   else
175     perpHlow(k)=0;
176   end
177 end
178
179 %Compute and sum the individual contributions
180 figure
181 for k=1:ncontrib  %loop on side chains
182   spind=0;
183   for l=1:2    % loop on H/D forms
184     for m=1:npH(k)   %loop on pH forms
185       coef=naa(k)/naatot;
186       if m==1
187         coef=coef*(100.-perpHlow(k))/100;
188       else
189         coef=coef*perpHlow(k)/100;
190       end
191       if l==1
192         coef=coef*(100.-perD(k))/100;
193       else
194         coef=coef*perD(k)/100;
195       end
196       if coef~=0
197         for n=1:nc(k,l,m) %loop on components of each side chain contribution
198           sg=fg(k,l,m,n)*ODmax(k,l,m,n)*exp(-(log(2.))*(2.*(xcm-freq(k,l,m,n))/FWHH(k,l,m,n)).^2);
199           sl=(1.-fg(k,l,m,n))*ODmax(k,l,m,n)*FWHH(k,l,m,n)^2./(FWHH(k,l,m,n)^2+4.*(xcm-freq(k,l,m,n)).^2);
200           spind=spind+coef*(sg+sl);
201         end
202       end
203     end
204   end
205   spres=spres+spind;
206   SideChain.spind(:,k)=spind';
207   line(xcm,spind,'Color',cust.sidechain(k).color,'LineWidth',3);
208 end
209 line(xcm,spres,'Color',[0 0 1],'LineWidth',3);
210 set(gca,'XDir','reverse'); xlabel('Wavenumber / cm^-^1', 'FontSize',16,'FontWeight','b'); ylabel('Absorbance', 'FontSize',16,'FontWeight','b');
211 tt=get(gca,'XTick');  set(gca,'XTick', tt, 'FontWeight','bold','FontSize',16); set(gca,'XLim',[xmin xmax]);
212 legend ('Asp','Glu','Tyr','His','Phe','Gln','Asn','Arg','Lys','C-term','N-term','SUM');
213
```

Table S1. Amino acid side chain band parameters for computing their contribution between 1720 and 1480 cm^{-1}. Lines 1-130 contain the parameters explained in details for Asp (lines 4-14). Parameters come from Wolpert and Hellwig (Wolpert and Hellwig 2006). Most of the band width come from Venyaminov and Kalnin (Venyaminov and Kalnin 1991). Where indicated in red, information has been obtained from Ramelow et al. (Rahmelow et al. 1998) or is deriving from personal observations as described in the paper (lines 23 and 35). Lines from 133 to 211 use the parameters to compute the contribution of each side chain and their sum and plot them. The entire text from line 1 to 211 can be pasted in Matlab as such.

References

Rahmelow K, Hubner W, Ackermann T (1998) Infrared absorbances of protein side chains. AnalBiochem 257:1–11

Venyaminov SYY, Kalnin NN (1991) Quantitative IR spectrophotometry of peptides compounds in water (H2O) solutions. I. Spectral parameters of amino acid residue absorption band. Biopolymers 30:1243–1257

Wolpert M, Hellwig P (2006) Infrared spectra and molar absorption coefficients of the 20 alpha amino acids in aqueous solutions in the spectral range from 1800 to 500 cm(-1). Spectrochim Acta A Mol Biomol Spectrosc 64:987–1001. https://doi.org/10.1016/j.saa.2005.08.025

Table S2: SVM, PLS and ASLR secondary structure prediction results

	cross-validation								Kennard-Stone							
	raw spectra				aa corrected				raw spectra				aa corrected			
**SVM**	STDDEV^{REFCV}	RMSECV	ζ^{CV}	r	STDDEV^{REFCV}	RMSECV	ζ^{CV}	r	STDDEV^{REFKS}	RMSEKS	ζ^{KS}	r	STDDEV^{REFKS}	RMSEKS	ζ^{KS}	r
dH	18.27	6.17	2.96	0.94	18.27	5.99	3.05	0.94	21.75	6.98	3.12	0.94	21.75	7.21	3.02	0.94
dE	13.67	5.37	2.55	0.92	13.67	5.02	2.73	0.93	15.15	6.2	2.44	0.92	15.15	5.44	2.79	0.93
dG	2.66	2.67	1	-0.27	2.66	2.65	1	0.02	2.55	2.54	1	-0.27	2.55	2.58	0.99	0.02
dT	4.09	4.11	0.99	-0.62	4.09	3.97	1.03	0.28	5.91	5.92	1	-0.62	5.91	6	0.99	0.28
dB	1.33	1.23	1.08	0.36	1.33	1.22	1.09	0.38	1.55	1.45	1.07	0.36	1.55	1.48	1.05	0.38
dS	4.39	3.48	1.26	0.62	4.39	3.44	1.28	0.63	6.36	4.55	1.4	0.62	6.36	4.43	1.43	0.63
d-	5.82	4.77	1.22	0.6	5.82	4.23	1.37	0.69	8.44	6.74	1.25	0.6	8.44	6.88	1.23	0.69
dOthers	10.26	6.16	1.67	0.81	10.26	5.95	1.72	0.81	15.09	9.41	1.6	0.81	15.09	10.62	1.42	0.81
**PLS**	STDDEV^{REFCV}	RMSECV	ζ^{CV}	r	STDDEV^{REFCV}	RMSECV	ζ^{CV}	r	STDDEV^{REFKS}	RMSEKS	ζ^{KS}	r	STDDEV^{REFKS}	RMSEKS	ζ^{KS}	r
dH (4 LVs)	18.27	6.3	2.9	0.94	18.27	6.82	2.68	0.93	21.75	6.52	3.33	0.95	21.75	7.56	2.88	0.94
dE (3LVs)	13.67	5.61	2.44	0.91	13.67	5.11	2.67	0.93	15.15	6.28	2.41	0.92	15.15	5.65	2.68	0.94
dG (5 LVs)	2.66	2.87	0.92	0	2.66	2.91	0.91	-0.01	2.55	3.51	0.73	-0.17	2.55	4.31	0.59	-0.39
dT (5 LVs)	4.09	4.37	0.94	0.11	4.09	4.28	0.96	0.19	5.91	5.61	1.06	0.27	5.91	6.19	0.96	-0.02
dB (5 LVs)	1.33	1.29	1.03	0.33	1.33	1.34	0.99	0.23	1.55	1.74	0.89	0.02	1.55	1.87	0.83	-0.18
dS (3 LVs)	4.39	3.81	1.15	0.51	4.39	3.79	1.16	0.52	6.36	5.22	1.22	0.61	6.36	5.2	1.22	0.58
d- (5 LVs)	5.82	4.83	1.2	0.57	5.82	4.89	1.19	0.56	8.44	7.33	1.15	0.47	8.44	7.43	1.14	0.45
dOthers (5 LVs)	10.26	7.9	1.3	0.64	10.26	7.66	1.34	0.67	15.09	10.62	1.42	0.79	15.09	10.53	1.43	0.81
**ASLR**	STDDEV^{REFCV}	RMSECV	ζ^{CV}	r	STDDEV^{REFCV}	RMSECV	ζ^{CV}	r	STDDEV^{REFKS}	RMSEKS	ζ^{KS}	r	STDDEV^{REFKS}	RMSEKS	ζ^{KS}	r
dH	18.27	5.75	3.18	0.95	18.27	6.48	2.82	0.93	21.75	6.05	3.6	0.96	21.75	7.43	2.93	0.94
dE	13.67	5.42	2.52	0.92	13.67	4.97	2.75	0.93	15.15	5.95	2.55	0.93	15.15	5.05	3	0.95
dG	2.66	2.57	1.03	0.2	2.66	2.56	1.04	0.2	2.55	2.43	1.05	0.36	2.55	2.77	0.92	0.16
dT	4.09	4	1.02	0.24	4.09	3.92	1.04	0.3	5.91	5.57	1.06	0.34	5.91	5.72	1.03	0.25
dB	1.33	1.19	1.11	0.38	1.33	1.25	1.06	0.25	1.55	1.62	0.96	0.19	1.55	1.58	0.98	0.11
dS	4.39	3.16	1.39	0.69	4.39	3.23	1.36	0.67	6.36	4.43	1.43	0.75	6.36	4.7	1.35	0.72
d-	5.82	4.5	1.29	0.64	5.82	4.52	1.29	0.63	8.44	6.5	1.3	0.66	8.44	6.15	1.37	0.73
dOthers	10.26	7.25	1.42	0.71	10.26	7.09	1.45	0.72	15.09	10.51	1.44	0.8	15.09	10	1.51	0.81

Table S2: characterization of models based on SVM (top), PLS (middle) and ASLR (bottom) for the prediction of DSSP-defined secondary structure content in cSP92 protein FTIR spectra. The left part of the table reports results obtained in leave-one-out cross-validation mode, the right part results obtained on the Kennard-Stone subset (1/3 of the proteins). STDDEV^{REF} is the standard deviation of the reference (DSSP) secondary structure content in the test set, RMSECV in the root mean square error in leave-one-our cross-validation and RMSEKS is the root mean square error for the Kennard-Stone subset. ζ^{CV} is defined as STDDEV^{REFCV}/RMSECV and ζ^{KS} as STDDEV^{REFKS} /RMSEKS; r is the correlation coefficient. The number of LVs used is indicated for PLS. For ASLR, 4 wavenumbers have been used. Spectra were either the original spectra (raw spectra, dark yellow) or the spectra corrected for amino acid side chain contributions (aa corrected, light yellow).
